# The Role of Staff-Assessed Care Quality in the Relationship between Job Demands and Stress in Human Service Work: The Example of Dentistry

**DOI:** 10.3390/ijerph191912795

**Published:** 2022-10-06

**Authors:** Işıl Karatuna, Mikaela Owen, Hugo Westerlund, Hanne Berthelsen

**Affiliations:** 1 Department of Psychology, Faculty of Social Sciences, Beykoz University, 34805 Istanbul, Turkey; 2 Centre for Workplace Excellence, University of South Australia, Adelaide, SA 5001, Australia; 3 Department of Psychology, Stress Research Institute, Stockholm University, 106 91 Stockholm, Sweden; 4 Centre for Work Life and Evaluation Studies (CTA) & the Faculty of Odontology, Malmö University, 205 06 Malmö, Sweden

**Keywords:** care quality, stress, job demands, dentistry, workplace

## Abstract

The aim of this study was to investigate staff-assessed care quality at the clinic as a predictor of stress and as a moderator between job demands (quantitative demands and role conflict) and stress among dental professionals as an example of human service workers. Cross-sectional questionnaire data from 1012 dental professionals (i.e., dentists, dental hygienists and dental nurses) working at 99 clinics were analysed by confirmatory factor analysis and a two-level hierarchical linear model. Stress, quantitative demands and role conflict were measured by the Swedish standard version of COPSOQ III and care quality was measured by three proprietary items. The results showed that staff-assessed care quality at the clinic was of importance for the individual workers’ experiences of stress. Furthermore, the staff’s joint assessment of the care quality at the clinic mitigated the negative effect of role conflict on stress among dental nurses. These results indicate that a high level of staff-assessed care quality at the clinic can contribute to reduced stress in dental professionals.

## 1. Introduction

Human service workers face many demands in their daily work that can lead to stress [1]. Dentistry is one of the core human service occupations where the work is notably stressful [2,3,4,5]. Job tasks that involve dealing with anxious patients, handling complicated treatments and meeting quality standards [6,7,8] can make dental work inherently demanding.

In dentistry, alongside other occupations in human services, emotional demands are an essential part of the work [9]. However, emotional demands do not occur in isolation; they need to be understood in the context in which they arise. That is, emotional demands might promote human service workers’ positive job attitudes when the level of parallel job demands is low. For example, Geisler et al. [10] found that emotional demands were positively linked to evaluations of the quality of the work performed and the meaning in work when other demands such as quantitative demands (e.g., high workload) and role conflict were low.

A high workload, also referred to as work pressure, is among the most common stressors reported in dentistry. For example, Ayers et al. [11] found that constant time pressure was one of the most frequently reported stressors among 700 dentists in New Zealand. More recently, Marklund et al. [12] found that nearly 50% of the dentists in Sweden experienced a high workload. This exposure to high quantitative demands in the workplace is of concern. With increasing exposure to time pressure and high workloads, dental professionals report higher levels of stress [4] and stress-related outcomes such as emotional exhaustion, depersonalization and cynicism [6,13].

Role conflicts are another common job demand in dentistry [14,15,16]. Role conflicts arise when engaging in job tasks that compete for the same resources (e.g., time and physical and/ or mental energy), or when expectations of the occupational role are inconsistent between the worker and the other members of the organization [17]. Work environments with high role conflict, as demonstrated by competing job tasks and inconsistent expectations, contribute to feelings of stress among dental professionals [14,15,18]. For example, in 1978 evidence was emerging that role conflicts predicted severe stress symptoms among dentists [14]. More recently, in a study conducted among oral-maxillofacial surgeons, Carneiro et al. [18] also found that high stress levels were associated with experiencing role conflicts, while an interview study emphasized role conflicts as an important concern in the work environment for dental hygienists [15].

The theory of Stress as Offense to Self (SOS) developed by Semmer et al. [19,20] can be used to understand the associations between role conflicts and stress in human service work. Semmer et al. [20] state that work tasks that interfere with the occupational role of the individual and thus are considered unnecessary or unreasonable (illegitimate), can be a source of stress. “Illegitimate tasks represent a special case of role conflict” [21] (p. 35) and they constitute a threat to the self because they violate the occupational role norms of an employee [21]. In accordance with SOS theory, preserving positive self-esteem is an important need and hence threats to self-esteem cause strain, whereas boosts to self-esteem foster well-being [20]. The theory distinguishes between personal self-esteem and social self-esteem. Personal self-esteem may be threatened by work aspects that are labelled as “stress through insufficiency”, while threats to social self-esteem may occur in terms of “stress as disrespect”. Stress through insufficiency includes personal feelings of success and failure in relation to one’s performance. If one cannot meet the criteria for good performance, then one will feel insufficient and thus be stressed due to a threat to self-esteem [20]. On the other hand, stress as disrespect contains messages of disrespect related to illegitimate behaviour, illegitimate tasks and illegitimate stressors. Therefore, if one is treated disrespectfully by others or assigned work tasks unrelated to the core of one’s occupation or exposed to a personally attributed stressful situation that prevents task completion, this is likely to be experienced as highly stressful [20]. For example, if one is assigned work tasks that signal disrespect, such as illegitimate tasks that violate the occupational role norms of an employee, then it is likely to create a threat to self, resulting in greater stress [21]. Furthermore, there is empirical evidence indicating that conflicting role expectations are associated with unreasonable tasks and thus are associated with higher stress [22].

In addition to role conflicts and quantitative demands, care quality is an important concept [23] associated with stress in healthcare settings [24,25]. According to one of the most-cited definitions, care quality represents “the degree to which health services for individuals and populations increase the likelihood of desired health outcomes and are consistent with current professional knowledge” [26] (p.11). Donabedian [27] states that there are two important aspects in assessing care quality, (1) technical care quality, which refers to the use of clinical knowledge and skills to promote the patient’s health, and (2) interpersonal care quality, which includes relationships between patients and health professionals. Care quality can be investigated both at the individual level (e.g., individuals’ perceptions of the quality they deliver) and at the group level (e.g., patient satisfaction, hospital mortality rates, infection rates and the aggregated perception of care provided by groups of individuals in teams, departments and organizations) [28]. Thus, the concept of care quality might differ depending on whether it is measured at the individual level with individually reported self-assessments, or at the group level with register-based measures of care quality, such as patient records, or with survey-based measures, such as patient satisfaction surveys [29]. In this study, we investigated the importance of staff-assessed care quality at the clinic for stress among the employees. Staff-assessed care quality (i.e., shared perceptions of the level of care quality at the clinic) is a relevant measure in dentistry because it is linked to the survival of dental fillings, a register-based measure of care quality [29]. So far, the association between care quality and stress symptoms has been examined mostly at the level of the individual care provider [24,25]. Thus, this study contributes to our understanding of the importance of the joint perception of care quality at the aggregated level (i.e., at the clinic) for individual staff members’ stress levels.

Previous research has shown a negative association between care quality and stress symptoms among healthcare employees. For example, a meta-analysis of 82 studies conducted among healthcare providers showed statistically significant negative relationships between stress symptoms (burnout) and care quality [25]. Most of the studies included in Salyers et al.’s study [25] had a cross-sectional design. However, in their longitudinal study Krämer et al. [30] found that high care quality predicted reduced time pressure among physicians. As such, we expected that staff-assessed care quality at the clinic would be of importance for stress among dental professionals. According to the stress through insufficiency facet of SOS, failing to meet performance standards would threaten one’s personal self-esteem and hence result in greater stress [20]. More specifically, health professionals’ perceptions of poor care quality at the clinic could impair their ability to feel capable of reaching their internal standards and consequently cause greater stress.

In accordance with the SOS theory, staff-assessed care quality might also have an impact on the relationship between job demands and stress. Because quality standards are central to human service professions and because a person’s profession is often central to their identity, quality deficits are likely to be threats to self. Accordingly, one might expect high quantitative demands and role conflicts to be perceived as less stressful when the care quality at the clinic is good. Therefore, we anticipated that working in a clinic with high levels of care quality would mitigate the detrimental effects of quantitative demands and role conflicts on stress. To date, we have found no research that has investigated the role of care quality in the relationship between job demands and stress in healthcare settings. The present study aims to address this gap.

### Aim/Hypotheses

The overall aim of the present study was to present the importance of staff-assessed care quality for stress among dental professionals. More specifically we aimed to investigate the role of staff-assessed care quality in the relationship between job demands and stress. Thus, the following hypotheses were stated:

**Hypothesis** **1a** **(H1a):** *Individually rated quantitative demands are positively correlated with individually rated stress;*

**Hypothesis** **1b** **(H1b):** *Individually rated role conflicts are positively correlated with individually rated stress;*

**Hypothesis** **2** **(H2):** *Staff-assessed care quality at the clinic is negatively correlated with individually rated stress;*

**Hypothesis** **3a** **(H3a):** *Staff-assessed care quality at the clinic mitigates the relationship between individually rated quantitative demands and stress;*

**Hypothesis** **3b** **(H3b):** *Staff-assessed care quality at the clinic mitigates the relationship between individually rated role conflicts and stress.*

**Figure 1:**
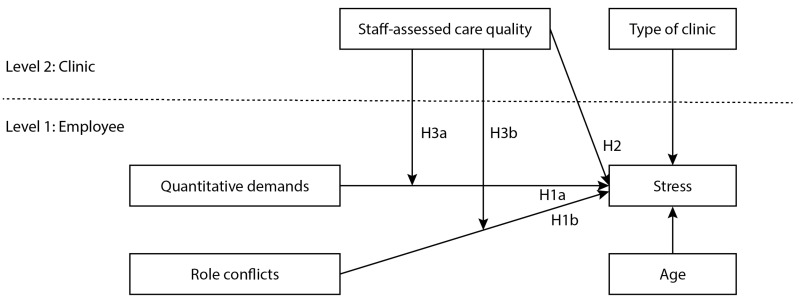
Conceptual model for the study.

While dental nurses in Sweden work under delegation, dentists and dental hygienists are licensed [31]. In his theory of human service organizations, Hasenfeld [9] emphasizes the uniqueness of client-worker relations and that human service organizations aim at improving the well-being of individuals (e.g., clients, patients and pupils) by altering or reshaping their personal attributes. Based on these characteristics, dentists and dental hygienists treating patients are core examples of human service workers, while dental nurses as auxiliary staff are not. Finally, dentists are considered a full profession and dental hygienists an emerging profession in Sweden [32]. Due to the theory of human service organizations [9] and the differences in the tasks and individual responsibilities among the occupational groups, the hypotheses were tested for each group separately. Figure 1 provides an overview of the conceptual study model.

## 2. Materials and Methods

### 2.1. Study Population

Data were collected between May 2014 and January 2015 in four regions of Sweden. An email including a personal login and password to an online questionnaire was sent to all staff employed at the regional Public Dental Health Service resulting in an overall response rate of 75% after two reminders. The inclusion criteria were non-managerial dental nurses, dental hygienists and dentists who had worked a minimum of one year at the clinic and a minimum of 20 h per week during the preceding three months. The clinics with at least three respondents meeting the inclusion criteria were included.

This resulted in a sample of 1012 respondents from 99 dental clinics (geographically separate dental practices where people conduct their daily work and share the same local management). Most of the respondents worked in general practice (80.3%), while every fifth respondent worked in specialized clinics (19.7%). Almost all the respondents had a permanent position (98.6%) and more than half (57.3%) worked full time, while only a few worked less than 30 h per week (5.7%). The characteristics of the study sample are presented in Table 1.

### 2.2. Measures

The following constructs were operationalized by the Swedish standard version of COPSOQ III [17,33]. Scale scores were calculated as the means of the items for each scale, including only those respondents who had answered at least half of the questions included in the scale.

*Quantitative demands* were measured by three items: (1) Is your workload unevenly distributed so it piles up?; (2) How often do you not have time to complete all your work tasks?; and (3) Do you get behind with your work?. The response options were: Always (100); Often (75); Sometimes (50); Seldom (25); and Never/hardly ever (0). Cronbach’s alpha for the scale = 0.82 in the current study.

*Role conflicts* were measured by three items: (1) Are contradictory demands placed on you at work?; (2) Do you sometimes have to do things which ought to have been done in a different way?; and (3) Do you sometimes have to do things which seem to be unnecessary?. The response options were: To a very large extent (100); To a large extent (75); Somewhat (50); To a small extent (25); and To a very small extent (0). Cronbach’s alpha for the scale = 0.60.

*Stress* was measured by three items with an introduction—These questions are about how you have been during the last four weeks: (1) How often have you had problems relaxing?; (2) How often have you been irritable?; and (3) How often have you been tense?. The response options were: All the time (100); A large part of the time (75); Part of the time (50); A small part of the time (25); and Not at all (0). Cronbach’s alpha for the scale = 0.89.

*Staff-assessed care quality at the clinic* was measured by three items [34]: (1) Are you satisfied with the quality of the work performed at your clinic? and a battery of two items with the introduction: To what extent do you find that the following issues characterize your clinic?; (2a) Is the quality of communication with patients good?; and (2b) Is the quality of the actual treatment of patients good?. The response options were: To a very large extent (100); To a large extent (75); Somewhat (50); To a small extent (25); and To a very small extent (0). A mean scale score was calculated for respondents who had answered at least two items. Individual-level scale scores were aggregated to an average scale score for each clinic. Cronbach’s alpha for the scale = 0.81.

As work experience is of importance for stress, the practice type (general or specialized practice) and the age of the respondent were included as covariates in the model.

### 2.3. Data Analyses

A confirmatory factor analysis (CFA) was conducted using IBM SPSS v26 and multigroup (CFA) in AMOS v26 for evaluating measurement invariance across occupational groups. Several indices were used to examine the overall fit of the model to the data. The χ2 test and root mean square error of approximation (RMSEA) were used as absolute goodness-of-fit indices. RMSEA values below 0.05 indicate a good fit, 0.06–0.08 a reasonable fit, 0.08–0.10 a mediocre fit and >0.10 a poor fit. In addition, the comparative fit index (CFI) and Tucker-Lewis index (TLI) were used as relative goodness-of-fit indices. The classical criterion for these two indices suggests that values over 0.90 or over 0.95 indicate a good model fit [35].

ANOVA tests with LSD and Tamhane’s T2 Post Hoc tests for multiple comparisons were used for analysing differences between occupational groups.

To justify aggregating individual-level scales scores for quality of care into an average clinic score (staff-assessed care quality), the intra-class correlation coefficients (ICC(1) and ICC(2)) were calculated. ICC(1) represents the amount of variance in the employees’ responses that can be explained by the clinic they work in [36,37,38,39] and ICC(1) values of 0.05 can be considered as a small to medium effect while higher values indicate stronger effects, i.e., a larger proportion of the variance is explained by the workplace [39]. ICC(1) values applied in field research of organizations are typically up to a maximum of 0.20 [37]. ICC(2) is an estimate of the reliability of the aggregated group means [36,37,38] and values <0.5 indicate poor reliability, 0.5–0.75 indicate moderate reliability and >0.75 indicate good reliability of group-level means [40]. 

A two-level hierarchical linear model using restricted maximum likelihood estimation with the individual employees (N = 1012) nested within dental clinics (N = 99) was analyzed using the R packages lme4 (v1.1-29), lmerTest (v3.1-3) and jtools (v2.20) (Figure 1). First, we tested an empty model containing only the random intercept (Model 1), which allowed us to compute the ICC(1) significance for stress. In Model 2, we entered the individual-level predictors (quantitative demands and role conflicts) of stress and the covariate (age) and at the clinic level we entered staff-assessed care quality and type of practice. The individual-level predictors were centered around the group mean and the staff-assessed care quality at the clinic level was centered around the grand mean. The tasks and responsibilities differed substantially for the different occupational groups; therefore, the models were tested for each group separately. We compared subsequent goodness-of-fit of models using the model fit indices log-likelihood and Akaike’s information criterion (AIC).

## 3. Results

First, we tested a confirmatory factor analytic (CFA) model with four factors—quantitative demands, role conflicts, staff-assessed care quality and stress—with each comprised of three items at the individual level. Across all items, the assumption of normality was met with no substantial kurtosis or signs of multivariate outliers. The proposed CFA model was a good fit to the data, as all model fit indices met their cut-off criteria (χ2(48) = 128.994, *p* < 0.001; CFI = 0.98; TLI = 0.97 and RMSEA = 0.044). The items loaded on the factors as expected, with factor loadings ranging from 0.55 to 0.92 (0.70–0.82 for quantitative demands, 0.55–0.62 for role conflicts, 0.79–0.92 for stress and 0.64–0.91 for care quality). The divergent validity of the four latent factors was corroborated (intercorrelations ranging from −0.23 to 0.54 in the expected directions). Next, we performed a configural invariance test by analysing a freely estimated model (unconstrained model) across two groups of respondents based on their occupation (dental nurses vs. dental hygienists and dentists). We obtained an excellent goodness of fit (χ2(96) = 168.801, *p* < 0.001; CFI = 0.98; TLI 0.98 and RMSEA = 0.029) thus corroborating the configural invariance. Comparing the measurement model (constraining all measurement weights across groups) to the unconstrained model corroborated the metric invariance between the groups (*p* = 0.56).

We found sufficient variation attributable to clinics (ICC(1) = 0.15) and moderate reliability (ICC(2) = 0.62) to justify aggregation of care quality to an average clinic score based on the entire staff group for inclusion at level 2 in the hierarchical linear models.

Significant differences in mean scale scores were seen depending on the occupational group (Table 2). Dentists reported on average higher levels of quantitative demands, role conflicts and stress than dental nurses and hygienists. Dental nurses reported higher care quality than dental hygienists and dentists.

Table 3 shows the results of multilevel regression analyses testing the hypotheses for dental nurses and dentists, separately. The results of Model 1 (null model without predictors and confounders) showed substantial between-clinic variation for stress (ICC(1) = 0.12 (*p* < 0.001) for dental nurses and 0.18 (*p* < 0.001) for dentists). For dental hygienists, who in general work more as sole practitioners, the ICC(1) was 0.01 (*p* = 0.19) indicating that there is no shared variance in stress to explain at the clinic level for this group. Thus, further analysis using a multilevel model was not warranted for the group of dental hygienists. The variables of interest entered in Model 2 contributed to explaining significant variance in stress (*p* < 0.001), resulting in an improved AIC and indicating a better model fit. As anticipated, quantitative demands and role conflicts were positively related to stress, while age and staff-assessed care quality at the clinic were negatively related to stress for both dental nurses and dentists.

**Figure 2:**
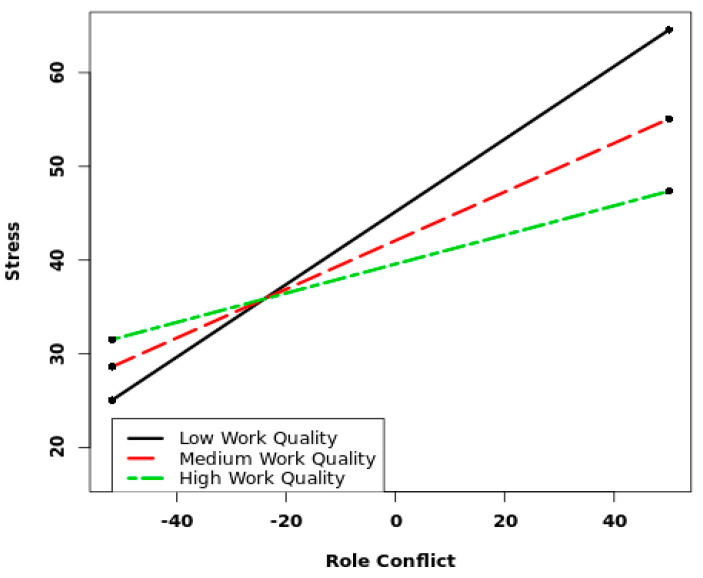
The moderation of staff-assessed care quality on the association between role conflict and stress among dental nurses.

**Figure 3:**
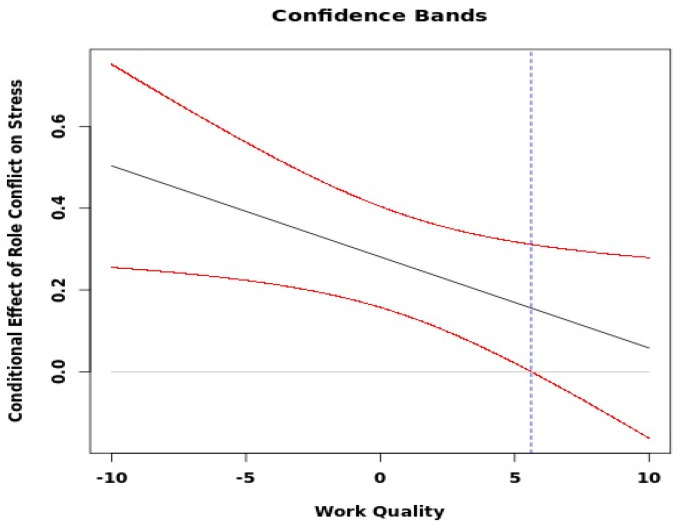
The conditional impact of role conflict on stress by staff-assessed care quality for dental nurses.

In Model 3 (Table 3) we included the cross-level interaction terms for staff-assessed care quality to test for moderation in the relationship between job demands and stress. The overall model fit was similar and all direct paths showed stable results, except for the association between role conflicts and stress that became non-significant for dentists. The care quality at the group level buffered the direct path from role conflicts to stress, though only for dental nurses and no other moderations were found. Figure 2 illustrates that as scores increased on care quality, the negative impact of role conflicts on stress decreased for dental nurses. Figure 3 shows that when care quality was high (75th percentile score) the slope between role conflict and stress was not significant. However, when care quality was low (25th percentile) or medium (50th percentile) there was a significant effect of role conflict. To sum up, with increasing levels of staff-assessed care quality, the negative impact of role conflict on stress decreased for dental nurses.

The type of practice (generalized or specialized) did not significantly impact stress levels in any of the tested models. Notably, across models, the care quality at the clinic level had the strongest association with stress as experienced by the individual care provider. This association between care quality and stress appeared to be stronger for dentists than for dental nurses. For each 10-point higher average level of care quality at the clinic, stress among dental nurses was reduced by 5 points and among dentists by 13 points on a scale of 0–100. Independently of occupation, however, significant variance of an equal size was left to be explained in stress (around 10% variance attributed to clinics) for both occupational groups.

Overall, the results of our analyses showed that individually rated quantitative demands and role conflicts were positively correlated and staff-assessed care quality at the clinic was negatively correlated with stress among dental professionals, confirming our Hypotheses 1a, 1b and 2. Moreover, the staff-assessed care quality at the clinic only mitigated the association between individually rated role conflicts and stress among dental nurses, thus partially supporting Hypothesis 3b and rejecting Hypothesis 3a. For an overview of the hypotheses and results, see Table 4.

## 4. Discussion

The main findings of this study supported our hypothesis that higher job demands are associated with higher levels of self-reported stress, while higher staff-assessed care quality at the clinic was associated with lower self-reported stress. We also found partial support for the hypothesized moderating effects of staff-assessed care quality.

The finding that higher levels of quantitative demands and role conflicts were associated with higher levels of stress among dental professionals is in line with previous studies investigating the sources of stress in dentistry [5,11,12,14,18]. For example, Wilson et al. [5] reported that running behind schedule (quantitative demand) is a major job stressor among general dental practitioners in England and Wales. Likewise, Carneiro et al. [18] found that stress levels of dentists increased with increasing job demands such as role conflicts.

The better the joint staff-assessed care quality was at the clinic, the lower the stress among dental professionals. This finding adds to previous research measuring care quality in healthcare settings based on staff perceptions of their own performance [30,41]. A reason that employees consider poor care quality at the clinic as stressful could be that perceived quality deficits at the clinic might prevent them from meeting their internal standards and thus might threaten their sense of being a competent person [21]. For example, Andrews et al. [42] found that nurses perceived that the inability to offer comprehensive and high-quality nursing care was associated with a loss of self-esteem. Semmer et al. [19] argued that anything that constitutes a threat to self-esteem is considered to play a major role in the experience of stress. Therefore, poor care quality at the clinic might threaten dental professionals’ self-esteem and thus cause greater stress. Moreover, in human service work such as dentistry, the importance of the moral foundation of the work is emphasized [9] and employees are generally guided by an ambition to provide high-quality work [43]. Accordingly, working in a clinic with high-quality standards might also boost self-esteem and lead to lower stress among employees.

Interestingly, we found that staff-assessed care quality at the clinic was more strongly linked to stress for dentists than for dental nurses. This may be due to the fact that dentists hold the overall responsibility of the work conducted by the team, while dental nurses, as auxiliary staff, do not have a corresponding direct responsibility for patients [44]. This makes it likely that dentists feel greater stress with regard to making mistakes due to quality deficits at the clinic and, therefore, are also more focused on ensuring the quality of work is satisfactory. In line with this, being responsible for a patient’s oral situation, always having to deliver perfect quality and work tasks where the risk of making a mistake is higher are strongly related to stress among dentists [45]. The limited research that has been conducted in relation to dental nurses directs attention to different kinds of stressors in addition to workload, such as the relationship with the dentist and lack of recognition [3,46,47], income level [3,47] and too few opportunities for professional development [3].

The results of our analyses provided only partial support for the moderating role of staff-assessed care quality at the clinic in the relationships between job demands and stress. For dental nurses, working in a clinic with high staff-assessed care quality buffered the negative effect of role conflicts on stress. This may be because experiencing role conflicts due to being assigned contradictory, illegitimate tasks might distract one’s attention from the primary work tasks and could inhibit accomplishing the legitimate tasks, and thus result in stress [19,20]. This may also be because working in a high-quality clinic may help employees to reach their goals, and thus to achieve a positive evaluation of themselves. This positive evaluation in turn might lead to a re-appraisal of stressors [48]. Accordingly, dental nurses might appraise their ability to manage the role conflicts based on the fulfilment gained from successful performance due to the high care quality at the clinic. However, the buffering effect of quality of care in the relationship between role conflict and stress was not significant for dentists. An explanation could be that dental nurses to a higher extent than dentists are assigned illegitimate tasks based on their position in the hierarchy.

Contrary to our expectations, care quality did not buffer the association between quantitative demands and stress for any of the groups. Previous research showed that quantitative demands (e.g., time pressure) represent one of the most common stressors among dental professionals [3,6,11,49]. Therefore, our findings may imply that the overall level of quantitative demands in dentistry is so high that it is distressing, even in the presence of good care quality at the clinic.

The findings of our study must be considered in the light of some limitations. First, the data are cross-sectional, which means that it is not possible to draw causal conclusions. Second, there is only limited generalizability of the findings because our sample included only dental professionals from Swedish public dental clinics. Third, there was a rather low Cronbach’s alpha score for the role conflict scale, suggesting that the results in relation to this scale should be interpreted with caution. Finally, the analyses are based on data collected during 2014–2015. However, no large reforms or changes in dental education or in regulations have taken place in recent years. On the other hand, a major strength of our study is that we used an aggregated measure of care quality. This decreased the risk of the common method bias stemming from self-reported data and thus provided a more objective estimate of the level of care quality.

Overall, the findings of the present study corroborate previous studies demonstrating the stressful working conditions in dentistry. However, in addition to the well-known stressful work characteristics of dentistry, the present study highlighted the importance of care quality at the clinic by demonstrating that low staff-assessed care quality is strongly linked to stress in dentistry and that the negative impact of role conflicts on stress decreased among dental nurses in the presence of high care quality at the clinic. Moreover, this study contributes to the existing literature by supporting the idea that staff-assessed care quality at the clinic might be a predictor of employee outcomes such as well-being. In that regard, some suggestions for future research can be offered. First, there is a need for longitudinal studies to reach conclusions about the causality between staff-assessed care quality and stress. Second, future research should include samples from other professional groups and settings to increase the understanding of the staff-assessed quality of the work performed in human service work.

## 5. Conclusions

The present study indicates that the staff’s assessment of the quality of care delivered at the clinic is important for self-reported stress in dentistry and that it may buffer the negative impact of role conflict on stress. Thus, our study findings suggest that it may be beneficial to integrate a focus on opportunities for delivering good care quality in the management of occupational health and safety for healthcare workers.

## Figures and Tables

**Table 1:** Participant demographics (*n* = 1012).

	*N*	*%*	*Mean (SD)*
Gender			
Women	936	92.5	
Men	76	7.5	
Age (years)			48.1 (11.3)
Weekly hours with patient contact			29.9 (9.2)
Job profile			
Dental nurses	575	56.8	
Dental hygienists	200	19.8	
Dentists	237	23.4	

**Table 2:** Mean, standard deviations (SD) for study variables based on occupational group.

*Scale*	*Occupation*	*Mean (SD)*	*p*	Post Hoc Tests *
Quantitative demands	1. Dental nurses	43.2 (17.8)	≤0.001	
	2. Dental hygienists	45.5 (16.5)		3 > 2
	3. Dentists	54.5 (20.0)		3 > 1
Role conflicts	1. Dental nurses	33.9 (17.4)	≤0.001	
	2. Dental hygienists	36.4 (16.2)		3 > 2
	3. Dentists	44.2 (16.6)		3 > 1
Staff-assessed care quality	1. Dental nurses	83.0 (13.8)	≤0.001	1 > 3
	2. Dental hygienists	80.5 (13.2)		1 > 2
	3. Dentists	78.3 (14.4)		
Stress	1. Dental nurses	28.3 (24.2)	≤0.001	
	2. Dental hygienists	31.8 (24.2)		3 > 2
	3. Dentists	38.1 (24.7)		3 > 1

Note. * Only significant mean differences between the groups are reported here (*p* ≤ 0.05).

**Table 3:** Multi-level linear regressions testing the associations of quantitative demands, role conflicts and staff-assessed care quality at the clinic with stress, for dental nurses (*n* = 567) and dentists (*n* = 235), controlling for age and kind of clinic.

	Dental Nurses (N = 567/99)	Dentists (N = 235/90)
	B (SE)	B (SE)
**Model 1 (empty model)**		
*Intercept*	28.35 (1.32) ***	37.65 (1.91) **
*ICC*	*0.12*	*0.18*
*AIC*	*5275.8*	*2180.6*
**Model 2 (direct paths model)**		
Staff-assessed care quality (level 2)	−0.54 (0.18) **	−1.27 (0.26) ***
Specialized practice (versus general) (level 2)	−3.27 (3.12)	8.05 (4.56)
Age (level 1)	−0.28 (0.10) **	−0.40 (0.12) ***
Role conflicts (level 1)	0.26 (0.06) ***	0.26 (0.12) *
Quantitative demands (level 1)	0.22 (0.06) ***	0.27 (0.10) **
*Intercept*	43.69 (5.38) ***	52.97 (5.28) ***
*ICC*	*0.10*	*0.10*
*AIC*	*5161.55*	*2134.15*
**Model 3 (moderated model)**		
Staff-assessed care quality (level 2)	−0.54 (0.18) **	−1.27 (0.26) ***
Specialized practice (versus general) (level 2)	−3.28 (3.13)	7.93 (4.57)
Age (level 1)	−0.28 (0.10) **	−0.39 (0.12) **
Role conflicts (level 1)	0.28 (0.06) ***	0.24 (0.13)
Quantitative demands (level 1)	0.21 (0.06) ***	0.27 (0.10) **
Staff-assessed care quality * Role conflicts	−0.02 (0.01) *	0.01 (0.02)
Staff-assessed care quality * Quantitative demands	−0.00 (0.01)	−0.02 (0.02)
*Intercept*	*43.39(5.37)*	*52.49(5.32)*
*ICC*	*0.10*	*0.10*
*AIC*	*5174.90*	*2149.26*

*** *p* < 0.001; ** *p* > 0.01; * *p* > 0.05. B: unstandardized linear regression coefficient, SE: standard error, ICC: intraclass correlation coefficient and AIC: Akaike information criterion.

**Table 4:** Results of the hypotheses for dentists and dental nurses.

Model	Hypotheses		Dental Nurses	Dentists
**Model 2** (Direct paths model)	1a	Individually rated quantitative demands are positively correlated with individually rated stress	Supported	Supported
	1b	Individually rated role conflicts are positively correlated with individually rated stress	Supported	Supported
	2	Staff-assessed care quality at the clinic is negatively correlated with individually rated stress	Supported	Supported
**Model 3 model** (Moderated model)	1a	Individually rated quantitative demands are positively correlated with individually rated stress	Supported	Supported
	1b	Individually rated role conflicts are positively correlated with individually rated stress	Supported	Not supported
	2	Staff-assessed care quality at the clinic is negatively correlated with individually rated stress	Supported	Supported
	3a	Staff-assessed care quality at the clinic mitigates the relationship between individually rated quantitative demands and stress	Not supported	Not supported
	3b	Staff-assessed care quality at the clinic mitigates the relationship between individually rated role conflicts and stress	Supported	Not supported

## Data Availability

Given the restrictions from the ethical review board and considering that sensitive personal data are handled, we are not able to make the data freely available. Researchers could contact us for specific supplementary analyses if needed or for a potential collaboration for access to data, though a supplementary ethical approval would be needed.
